# Case of Occlusion of the Vagina

**Published:** 1854-06

**Authors:** Charles H. Baker


ART. III.—*Case of Occlusion of the Vagina.* Reported to the Buffalo Medical Association, by CHARLES H. BAKER, M. D.

Was called, 11 P. M., Jan'y 30, to see Mrs. J. D., in her first labor—aged 20, of good constitution and plethoric habit. Had been married 18 months, and expected to have been confined by the 25th ult. Labor had been in progress since 7 P. M. Pains regular, severe, and apparently effective.

On examination, found the vagina quite contracted and terminating in a cul-de-sac, from two to three inches within the canal. The finger could detect no opening through the bottom of the sac, but by pressing upon it the os uteri was felt, (the septum intervening) dilated to nearly the size of a half dollar—the vertex presenting, and occupying the superior strait of the pelvis. On inquiring of the husband, was informed that he had never been able to effect an entrance during coition, and all that was possible to accomplish during an 18 months trial, was a deepening of the sac. Mrs. D. states that she has menstruated regularly since 16; the discharges escaping via naturalis; that she has never, within her recollection, had any soreness or inflammation of the parts. Up to nearly 3 A. M. of the 31st, the pains continued regular and effective; the head slowly advancing, and external orifice of vagina gradually dilating, when suddenly, the pains assumed the most agonizing character, occurring at intervals of one minute or less. She described the pains as "tearing her all to pieces, low down in the belly and end of the back," and although they retained this expulsive character, she could not "bear down" with them, but would toss wildly about, uttering the most piercing screams.

The pains now evidently producing more misery than benefit, at 3½ A. M. I administered a ½ grain of S. morph., and repeated the dose in ten minutes. At 4 A. M., there being no alteration of symptoms, gave another ½ grain morph., and dispatched a messenger for Dr. J. P. White, Prof. of Obst. in Buffalo University. Her pulse up to Dr. W.'s arrival had not gone above 86. Skin cool, but began to complain of great thirst, and soreness through the hypogastrium, and could not be raised to the sitting posture without producing the most intense suffering. As her strength still held good it was deemed advisable to trust a while further to the efforts of nature to overcome the obstruction, and, as her pains still continued their frequency and intensity, to repeat the anodyne.

Accordingly, at 6 A. M., 1 gr. morph. was given in injection. As no relief followed, this was repeated in half an hour, but with no better success.

Dr. W. had in the meantime left — to return at 8 A. M., prepared for any ulterior measures that might be necessary.

8½ A. M. Present Drs. White, Rochester, Hunt, Woods, and myself. The condition of parts not materially changed since Dr. W.'s departure. The os uteri and protruding bag of waters could be distinctly felt through the septum. The head could also be felt in the cavity of the pelvis — advancing with each pain against the septum, and receding with its departure. The tumor formed by the head could be as well reached through the rectum as the vagina, and the recto-vaginal septum seemed thinner than the occluding membrain. The pulsations of the fœtal heart were strong and distinct, beating 130 per minute, and showed the position to be, "the occiput to the left anteriorly." The pains were still very propulsive, and attended with exquisite pain.

Although the occluding membrane was not at this, or any other time, put forcibly on the stretch, it was evident that the unusual degree of pain depended on the resistance it offered. The adhesions were so near the os uteri, that the process of dilatation must have been obstructed by it sufficiently to account for the peculiar character of the pain.

The strength of patient was evidently failing, pulse 116. Drs. Rochester and Hunt examined, and concurred in the diagnosis, and the necessity for an operation.

9½ A. M. Patient was placed in the position for artificial delivery, and an examination made with the speculum. Not the slightest perforation, or appearance of a cicatrix was perceptible in the septum, which seems to be continuous, and identical in structure with the mucous membrane of the canal.

Not deeming it prudent to wait any longer, the patient was put under the influence of chloroform, and Dr. White proceeded to puncture the septum with Jobert's long scalpel for excising the lips of the os uteri. He then retracted the labia with the flat, bent specula, used for that purpose by Jobert, and these being confided to assistants, introduced a scalpel and enlarged the opening in all directions, when the chloroform was discontinued.

The liquor amnii now escaped, and the pains assumed a more distinct bearing down character, to which the patient, for the first time since about 3 A. M., lent her own assistance, and the head advanced gradually into the concavity of the sacrum.

11, A. M. The pulse becoming more feeble and the pains lessening, it was decided to apply the forceps, which was done by Dr. White, and the child (weighing 11 lbs.) delivered in a state of asphyxia. The mother was very much exhausted, but rallied under stimulants.

The warm bath being found insufficient for the recovery of the child, Dr. Hunt introduced Charriere's tracheal tube, and after keeping up artificial respiration for nearly an hour, circulation and respiration were fully and permanently established.

After delivery, patient rested entirely free from pain, and inclining to sleep, from which she was occasionally roused to partake of nourishing and stimulating drinks. Pulse 130. Enjoined perfect quiet. At 2 P. M. left her very comfortable.

8 P. M. Dr. Woods kindly visited the patient for me, and reports her as still comfortable. Pulse 128. Skin warm and moist. Had been no hemorrhage, although she had been, contrary to orders, removed to her bed. Complains of great soreness of external parts. Ordered saturnine lotion and cooling drinks.

Feb. 1st—10 A. M. Patient had a quiet night; slept well. Very little tenderness of abdomen. Pulse 100. Gentle perspiration. Has passed no urine. No uneasiness or pain around bladder.

Ordered solution of S. morph., $\frac{1}{8}$ gr., to be repeated as necessary.

During the night the child had convulsions, with free evacuations of bowels. Had a "fit" when I was present, accompanied apparently with griping. Had passed no urine. Ordered warm bath immediately, and 3 drops Spts. Nit. dulc. in a little sweetened water every two hours. The baths to be repeated whenever the fits occurred. At a subsequent day learned that one of the nurses, more officious than prudent, had given the child the evening previous, nearly a *tea-spoonful of Castor oil.*

2d—10 A. M. Had a comfortable day and night. Toward morning some pain in bladder, and the urine would escape in small quantities, producing great distress. Introduced catheter, and drew off nearly a pint of water. Parts considerably swollen and very sore. Secretion of milk has commenced. Appetite good. Pulse 96.

Morph. continued.

Child has had but two attacks since last reported; gaining strength, although not able to nurse. Passed urine freely. Nitre discontinued. It was now fed the mother's milk.

3d—10 A. M. Passed a sleepless night. Pulse 100. Breasts full and painful. No soreness or pain of abdomen; urine free; appetite good. At 2 A. M. had a severe chill, lasting nearly half an hour, followed by a profuse warm perspiration. Bears pressure on abdomen well. Tumefaction and soreness of parts nearly gone. Lochial discharge free.

Morph. continued.

4th — 2 P. M. Excepting a slight headache, patient is very comfortable. Pulse 96. Appetite good; bowels open. Gains strength fast.

Treatment continued.

From this date both mother and child recovered rapidly, with little necessity for medical aid; and at this time, May 1st, are both remarkably stout and healthy.

